# Application of Liquid Waste from Biogas Production for Microalgae *Chlorella* sp. Cultivation

**DOI:** 10.3390/cells11071206

**Published:** 2022-04-03

**Authors:** Egle Sendzikiene, Violeta Makareviciene

**Affiliations:** Faculty of Forest Sciences and Ecology, Agriculture Academy Vytautas Magnus University, Donelaicio Str. 58, LT-44248 Kaunas, Lithuania; violeta.makareviciene@vdu.lt

**Keywords:** *Chlorella* sp., biomass, cultivation, liquid waste after biogas production, glycerol, biomass yield, elemental composition

## Abstract

Microalgae biomass is a viable feedstock for a wide range of industries. Recently, there has also been interest in the ability of microalgae biomass applications for biofuel production. In the meantime, the cultivation of microalgae biomass requires high energy costs, and the application of microalgae for technical purposes is still problematic. A significant part of the cost of biomass arises from the nutrients used for cultivation. Chemical compounds included in the microalgae cultivation media can be replaced by suitable wastes containing nitrogen, phosphorus, and other elements. This could reduce the microalgae biomass cultivation price and allow cheaper biomass to be used for biofuel production. The aim of this work was to comprehensively investigate and optimize the growth process of microalgae using liquid waste (liquid waste after biogas production from sewage sludge and distillers’ grain) as a source of nitrogen and phosphorus, and technical glycerol as a carbon source. It was found that higher levels of waste in the cultivation media were found to inhibit the accumulation of microalgal biomass, with the optimum level corresponding to a nitrogen concentration of 0.08 g/L. The influence of technical glycerol from biodiesel production on the yield of microalgal biomass was investigated, and it was found that the addition of 6% glycerol allows an increase in the concentration of microalgal biomass in the cultivation media, from 18.1 to 20.6%.

## 1. Introduction

Recently, there has been increasing interest in the technical applications of microalgal biomass, including the production of biodiesel, biopolymers, and biogas [1]. The use of microalgae oil for biodiesel production is attractive because microalgae do not compete with oilseed crops for land area, they accumulate biomass rapidly and, under the right conditions, microalgae cells accumulate a large quantity of oil [2]. However, the accumulation of microalgal biomass requires nutrients.

In addition to carbon, the main macronutrients required for algal development are nitrogen and phosphorus [3]. Given that algae use nitrogen and phosphorus compounds as nutrients, and that these are liquid waste from biogas production, landfill leachate can become a water source for algae, and the NH_4_^+^, NO_3_^−^, NO_2_^−^, and PO_4_^3−^ ions contained therein can be used as nutrients for their cell growth and, thus, for pollutant removal. Microalgae can obtain nitrogen and phosphorus from the extracted microalgal biomass. In this way, microalgae can be cultivated using their oil, and the hydrothermally treated biomass can be returned to the microalgae cultivation process [4]. Growing microalgae in wastewater has a number of advantages, such as cost savings on chemicals used to prepare the microalgae cultivation media, wastewater treatment, and cheap microalgae biomass that can be used for technical applications, including biodiesel or biogas production. Studies have been conducted on the use of microalgae for the treatment of sewage wastewater [5,6] Sharma et al. investigated the potential of microalgae for the treatment of domestic wastewater [7]. Microalgae exhibited high purification efficiency in aquaculture wastewater and piggery wastewater [8,9], and have been used to treat cassava wastewater [10] and palm oil and starch processing wastewater [11,12].

Some researchers suggest the use of microalgae consortia for wastewater treatment. Silambarasan et al. [13] used a microalgae-based microalgae treatment system for the cultivation of a *Chlorella* sp. and *Scenedesmus* sp. consortium in diluted wastewater; they achieved not only a high biomass concentration but also a lipid content of 34.83%.

Udom et al. used wastewater centrate as the feed source for microalgae that were grown in a photobioreactor [14]. *Chlorella sorokiniana* microalgae were grown in photobioreactors using a liquid fraction of pig manure, obtaining a maximum dry biomass gain of 26.3 mg/L/day [15].

A mixotrophic cultivation method can be used for microalgae cultivation, where microalgae perform photosynthesis and use both organic and inorganic carbon as a carbon source for growth, and where the respiration and photosynthetic metabolism of microalgae occur simultaneously [16,17]. Low-cost sources of organic carbon are being sought to optimise the algal cultivation process under mixotrophic conditions.

Heredia-Arroyo et al. found that the addition of small amounts of glucose, sucrose, or maltose to microalgae nutrient media increases the rate of accumulation and yield of microalgal biomass [18]. This was supported also by Zhang et al., who investigated the mixotrophic cultivation of *Botryococcus brauni* using maltose, glucose, sucrose, lactose, glycerol, starch, and potassium nitrate as carbon sources, and found that the most efficiently used source was glucose [19]. Some authors have investigated the effectiveness of glycerol supplementation in microalgae cultivation and found that the addition of 10 g/L glycerol to the microalgae growth medium can increase the growth rate of microalgal biomass by up to 6.3 times [20].

Other researchers analysed glucose, glycerol, acetate, and glycine as carbon sources in the *Botryococcus brauni* cultivation process and estimated that the most effective one, glycine, with a content of 0.5 g/L, increases the biomass concentration from 1.49 to 2 g/L [21].

Zhao et al. used carbon dioxide to grow *Chlorella* sp., and found that the rate of carbon dioxide fixation was up to 5.39 times higher in closed-culture systems than in open ones [22]. Other researchers have found an effect of carbon dioxide on the growth of different microalgae species. *T. suecica* grows most efficiently when supplied with 15 and 5% CO_2_, with a biomass gain of 0.72 g/L and a CO_2_ biofixation of 111.26 mg/L/day. *Chlorella* sp. has a biomass gain of 0.64 g/L when supplied with 5 and 15% CO_2_, with a biofixation of 96.89 mg/L/day [23]. Carbon dioxide gas containing SO_x_ (up to 200 ppm) and NO_x_ (up to 150 ppm) has been found to be successfully used for microalgae cultivation [24,25]. This has been supported by other researchers who have evaluated the potential use of microalgae for biogas treatment [26].

The results show that a wide range of wastes can be used to grow microalgae, while carbon sources include carbon dioxide, carbohydrates, and other organic matter. Some researchers have used glycerol as a carbon source. However, pure glycerol has been used for research. The production of biodiesel produces technical glycerol as a by-product, the amount of which increases with increasing biodiesel volumes [27]. The purification of technical glycerol requires additional costs, so direct uses are being sought. One of these could be for the cultivation of microalgae, but technical glycerol contains a variety of impurities that could be harmful to microalgae. There are no research results on the use of technical glycerol as a carbon source and its effect on microalgae growth. The aim of our work was to comprehensively investigate and optimize the growth process of microalgae using liquid waste (liquid waste after biogas production from sewage sludge and distillers’ grain) as a source of nitrogen and phosphorus, and technical glycerol as a carbon source, in order to obtain the highest possible biomass gain in the shortest time.

## 2. Materials and Methods

The studies were carried out using *Chlorella* sp. green algae. They were grown under mixotrophic conditions, in the presence of nutrients of organic and inorganic origin in the cultivation media. To optimise the cultivation conditions, the microalgae were grown in conical flasks at room temperature, at 22 ± 2 °C, with magnetic stirring and illumination with fluorescent lamps emitting cold white light for 10 h/day. The culture volume was 1 L, and the light intensity was 100 µmol protons/m^2^s. Illuminance was measured with a data logger (model LI-1400) LI-190SA quantum sensor.

For the control studies, the microalgae were grown in BG11 cultivation media, which was prepared in laboratory conditions using distilled water and chemicals purchased from national chemical suppliers. The purity of the chemicals was at least analytical grade. The composition of the BG11 media is given in Table 1.

The liquid waste was autoclaved before incorporation into the cultivation media and filtered from hanging particles. The liquid waste was analysed for nitrogen content by a Kjeldahl apparatus, and a quantity of liquid waste was added to the BG11 cultivation media such that, when using waste feedstock as a nitrogen source, the cultivation media contained between 0.05 g/L and 0.14 g/L nitrogen. The liquid fraction of biogas production from distillers’ grain contained 610 mg/L total nitrogen and 95 mg/L total phosphorus, while the liquid fraction of biogas production from sewage sludge contained 980 mg/L total nitrogen and 69 mg/L total phosphorus.

Technical glycerol, a by-product of biodiesel production, was added to the cultivation media as an additional source of organic carbon. The efficiency of its application was analysed by adding between 2 and 10 g/L technical glycerol to the cultivation media.

In order to select the conditions under which the highest concentration of microalgae biomass is obtained, cultivation trials were carried out with the following nutrient composition:

- control—BG11: nitrogen concentration—0.12 g/L;
- liquid waste as a source of nitrogen + BG11: nitrogen concentrations from 0.05 g/L to 0.14 g/L;
- liquid waste + BG11 + technical glycerol: nitrogen concentration: 0.08 g/L; glycerol concentration from 2 g/L to 10 g/L.

The concentration of microalgae biomass was analysed by the spectrometric method using a Lambda 25 UV/Vis spectrophotometer. The optical density of the microalgal suspension was measured every 3 days of cultivation at 750 nm. From the optical density values, the concentration of microalgae biomass in the sample was determined using a calibration curve. The calibration curve was constructed from the values of the microalgae concentration determined by the weight method, and the optical density of the suspension was determined by the spectrometric method. For the determination of the microalgae concentration by weight, the suspension was centrifuged for 10 min at a speed of 12,000 min^−1^, and the biomass was washed with distilled water and dried at 105 °C to constant weight. The concentration of dry biomass (B) in the suspension was calculated according to Equation (1):

(1) $$B=\frac{m\times 100}{V}$$

where: 

*m*—mass of the dried sample, g;

*V* is the volume of the suspension taken for analysis, mL.

The microalgal biomass yield (g/L day) is calculated from the variation of biomass concentration per unit time. Biomass yield (*BI*) is calculated according to Equation (2):

(2) $$BI=\frac{X_{1}-X_{0}}{t_{1}-t_{0}}$$

Equation (3) was used to calculate the relative growth rate of microalgae (biomass gain per unit time (g/g d or 1/d)):

(3) μ=ln(X1X0)t1−t0

where *X*_1_ and *X*_0_ are the biomass concentrations (g/L) at days *t*_1_ and *t*_0_.

The elemental composition of the microalgae was analysed using a CHNS-O (Perkin-Elmer 2400) analyser after cultivation of the microalgae. Prior to analysis with the elemental composition analyser, the microalgae were centrifuged, and the resulting biomass was dried, washed with distilled water, and dried at 105 °C to constant weight. The microalgae cultivation conditions were optimised by assessing the biomass growth rate at different cultivation conditions and taking into account the elemental composition of the microalgal biomass.

All experiments were performed by three replications; as a final result, the mean value was used.

## 3. Results

### 3.1. Application of Liquid Waste from Biofuel Production for Microalgae Cultivation

**Figure 1:**
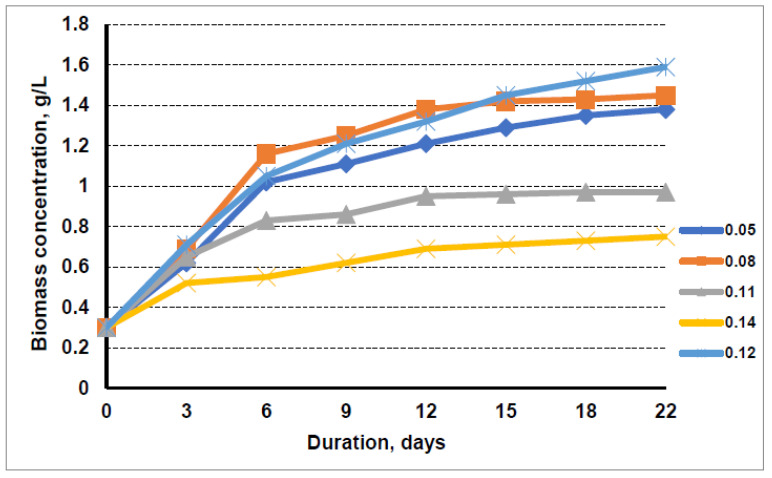
Growth dynamics of microalgae in nutrient medium with the liquid fraction after biogas production from distillers’ grain at nitrogen concentrations of 0.05 g/L, 0.08 g/L, 0.11 g/L, 0.14 g/L, and 0.12 g/L (control).

In order to investigate the suitability of liquid waste as a replacement for mineral nitrogen in microalgae growth media, studies were carried out on microalgae cultivation in BG11 growing media, where the nitrogen source was liquid waste, and the nitrogen concentrations were varied. Figure 1 shows the dynamics of biomass accumulation of microalgae when cultivated in a nutrient medium where the nitrogen source was replaced by the liquid fraction after biogas production from distillers’ grains. The composition of the liquid fraction after biogas production from distillers’ shavings was analysed and found to contain 660 mg/L total nitrogen and 407 mg/L ammonium nitrogen. Using this substrate, nutrient media with nitrogen concentrations ranging from 0.05 g/L to 0.14 g/L were prepared and experiments were carried out to grow microalgae by periodically measuring the biomass concentration in suspension. As a control for the cultivation of microalgae, conventional cultivation media BG11 was used. The results obtained are shown in Figure 1.

The data show that, in the initial phase, the accumulation of microalgal biomass was not very dependent on the nitrogen concentration in the cultivation media. The highest final concentration of 1.45 g/L was obtained when the microalgae were grown in 0.08 g/L nitrogen-containing media, which was prepared using the liquid fraction after biogas production from distillers’ grains. The lowest nitrogen concentration of 0.05 g/L in the cultivation media resulted in a lower growth intensity of microalgae, which resulted in a 1.38 g/L microalgae concentration in the suspension after 22 days of cultivation. The use of the higher nitrogen concentration on the accumulation of microalgal biomass at day 22 of cultivation showed a negative effect, compared with cultivation in BG media, where 1.59 g/L microalgae biomass was accumulated in the suspension. The cultivation of algae in a medium with a maximum nitrogen content of 0.14 g/L resulted in a microalgal biomass concentration of only 0.75 g/L, which was half that of the algae cultivated in a medium with 0.08 g/L nitrogen. Increasing the nitrogen concentration from 0.08 g/L to 0.11 g/L, i.e., the level of nitrogen typical of a conventional BG11 cultivation media, also significantly slowed down the accumulation of microalgal biomass. Only 0.97 g/L biomass concentration was obtained over 22 days, when algae was cultivated in medium containing 0.11 g/L nitrogen from distillers’ grains. The negative nitrogen effect of the liquid fraction after biogas production from distillers’ grains compared to the pure BG11 media can be explained by the fact that, in the BG11 media, all the nitrogen is in the nitrate form, which is taken up well and tolerated by microalgae. Meanwhile, the liquid fraction after biogas production from distillers’ grains contained relatively high levels of ammonium nitrogen.

**Figure 2:**
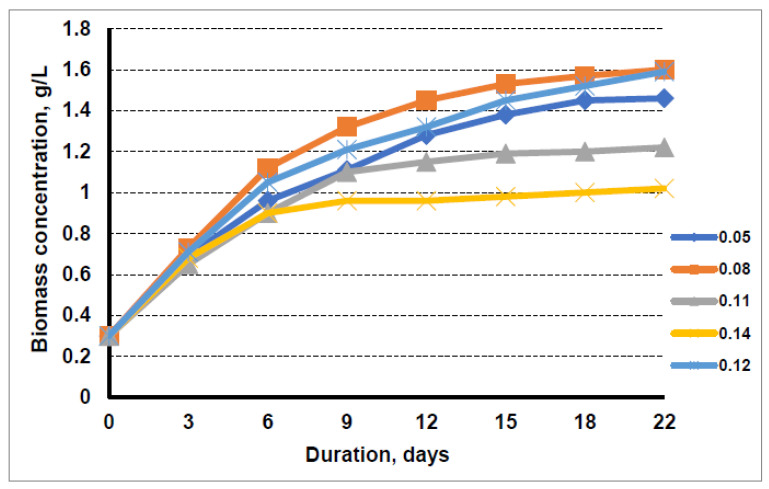
Growth dynamics of microalgae in nutrient medium with the liquid fraction after biogas production from sewage sludge at nitrogen concentrations of 0.05 g/L, 0.08 g/L, 0.11 g/L, 0.14 g/L, and 0.12 g/L (medium-control).

In order to assess the potential of suitable waste materials and to make microalgae cultivation cheaper, another substrate obtained in biogas production was tested: the liquid fraction from sewage sludge after biogas production. This was used to replace the nitrogen present in the conventional cultivation media BG11. The waste fraction contained 989 mg/L total nitrogen and 532 mg/L ammonium nitrogen. The nitrogen concentration variations in the microalgae cultivation media were the same as when using the liquid fraction after biogas production from distillers’ grains. The results obtained are presented in Figure 2. It can be seen that, again, the highest microalgae biomass yield was obtained using cultivation media containing a nitrogen concentration of 0.08 g/L. At the end of 22 days, the microalgae biomass concentration cultivating in the medium of this composition was the same as the cultivating microalgae in BG11 media. The highest biomass yield achieved was 0.125 g/L/day, and the highest relative growth rate was 0.198/day. Increasing the nitrogen content of the liquid fraction after biogas production from sewage sludge in the nutrient medium (nitrogen concentration above 0.08 g/L) resulted in a decreasing microalgal biomass yield, and at the highest studied nitrogen concentration of 0.14 g/L in the cultivation media, the microalgal biomass concentration was only 1.02 g/L over 22 days.

It should be noted that the growth rate of the algae in the medium with 0.05 g/L nitrogen was slower at the initial stage than in the medium with 0.08 g/L nitrogen, but by the 22nd day of cultivation in this medium, the concentration of microalgae biomass in suspension had reached a value similar to that in the medium with 0.08 g/L nitrogen, i.e., 1.6 g/L.

The negative impact of the higher liquid fraction on the growth of microalgal biomass can be explained, as in the case discussed above, by the fact that a large part of the nitrogen in the liquid waste is in the ammonium form, which is not easily tolerated by microalgae.

Microalgae can be cultivated in mixotrophic conditions using organic carbon sources [16,17,18]. In order to reduce the cost of growing microalgal biomass, cheaper sources of organic carbon must be sought. For our study, technical glycerol, which is produced during biodiesel production, was chosen. While the amount of technical glycerol has increased dramatically in Europe with the expansion of biodiesel production, the market demand for purified glycerol has remained the same, and biodiesel producers are looking for new uses of technical glycerol. The direct use of technical glycerol for microalgae cultivation would be one of the alternatives to the use of cheaper feedstocks containing organic carbon.

In view of the results obtained previously for the cultivation of microalgae using liquid waste, the cultivation media containing the optimum amount of liquid waste (nitrogen concentration of 0.08 g/L) were selected for the glycerol efficiency studies. The technical glycerol was added to the cultivation media with the liquid fraction after biogas production from distillers’ grain and sewage sludge. For comparison, tests were also carried out using the conventional microalgae cultivation media BG11. The composition of the technical glycerol was as follows: glycerol content—85.8%, water content—5.8%, content of free fatty acids—1.1%, and traces of methanol. According to the results of other studies, between 2 and 10% glycerol was added to the microalgae cultivation media. The accumulation of microalgal biomass was monitored for 22 days.

**Figure 3:**
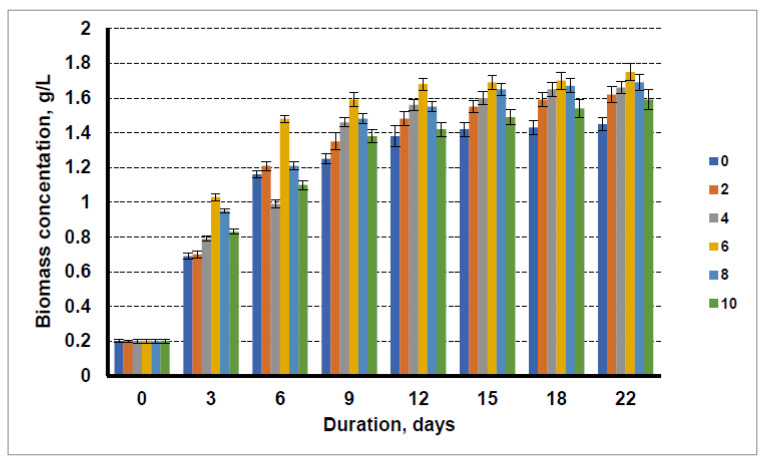
Growth dynamics of microalgae in the nutrient medium with the liquid fraction after biogas production from distillers’ grain with a nitrogen concentration of 0.08 g/L and glycerol concentrations of 0 g/L, 2 g/L, 4 g/L, 6 g/L, 8 g/L, and 10 g/L.

The results obtained using the cultivation media with the liquid fraction after biogas production from distillers’ grain and the sewage sludge are shown in Figure 3. It is clear that that using the cultivation media containing liquid waste after biogas production from distillers’ grain and the addition of technical glycerol increased the biomass yield of microalgae. The biomass yield increased by increasing concentration of technical glycerol in the cultivation media up to 6%, and at even higher concentrations, a lower biomass yield was obtained. The maximum biomass yield obtained for microalgae was 1.75 g/L. Even at a higher glycerol concentration of 10 g/L, the biomass yield of microalgae at the end of cultivation was higher (1.59 g/L) than that of the algae cultivated without the addition of glycerol (1.45 g/L). In this case, the glycerol additive increased the biomass yield by 20.6%.

**Figure 4:**
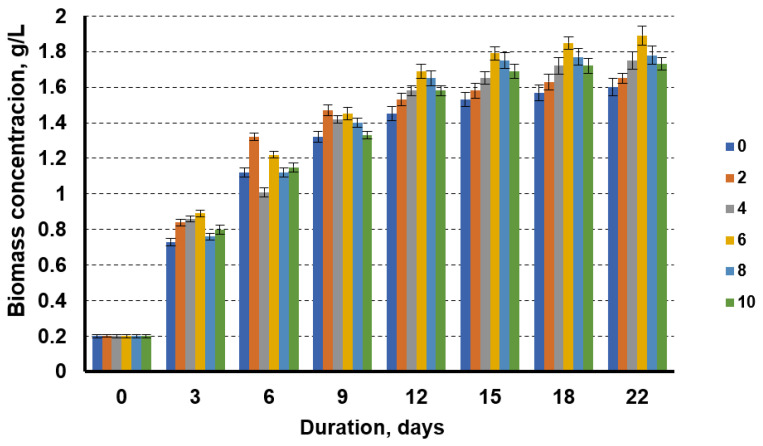
Growth dynamics of microalgae in the nutrient medium with the liquid fraction after biogas production from sewage sludge with a nitrogen concentration of 0.08 g/L and glycerol concentrations of 0 g/L, 2 g/L, 4 g/L, 6 g/L, 8 g/L, and 10 g/L.

Figure 4 shows the dynamics of the biomass accumulation of microalgae when cultivated in a medium where the nitrogen source was replaced by the liquid fraction of the biogas production from sewage sludge, with the addition of different amounts of technical glycerol. The data show that, also in this case, the addition of technical glycerol increased the biomass yield of the microalgae, which was also highest in the cultivation media, with 6 g/L technical glycerol. Increasing the glycerol content from 2 to 6% showed an increasing trend in the microalgal biomass yield; however, using the glycerol concentrations above 6%, a decrease in the microalgal biomass yield was observed. The lowest biomass concentration (1.6 g/L) was obtained when the microalgae were cultivated without the technical glycerol additive. At 6% glycerol in the cultivation media, the microalgae biomass concentration at day 22 of cultivation was 1.89 g/L. In this case, the addition of 6% technical glycerol increased the biomass yield by 18.1%.

### 3.2. Elemental Composition of Microalgae Biomass

In order to assess the influence of the composition of the cultivation media on the elemental composition of microalgal biomass, the composition of microalgal biomass cultivated in a media where the nitrogen source was replaced by two liquid fractions from biogas production from distillers’ grain, which varied in content, i.e., the concentration of nitrogen in the medium, ranged from 0.05 to 0.12 g/L (Table 2). For comparison, studies on the elemental composition of the biomass of microalgae cultivated in conventional media BG11 were carried out.

The data show that the carbon content of microalgal biomass is, in all cases, above 50%. The elemental carbon concentration in dry microalgal biomass varies between 53.8% and 56.8%. Moreover, it is not directly dependent on the nitrogen concentration in the microalgae cultivation media. The highest carbon concentrations were obtained when microalgae were cultivated in the BG11 media. A different situation was observed when analysing the nitrogen content of microalgae biomass. The nitrogen content of microalgae biomass varied from 6.98% to 7.77%. The results in Table 2 show that, as the nitrogen concentration in the cultivation media decreases, the microalgae accumulate less nitrogen in their cells. This can be explained by the fact that when nitrogen concentrations are lower, microalgae cells become stressed and protein production slows down. The data show that higher nitrogen accumulation in microalgal biomass occurs at higher nitrogen concentrations in the nutrient medium, but in this case, the yield of microalgal biomass is lower.

Another important element is phosphorus. The data in Table 3 show that the amount of phosphorus compounds accumulated in the cells of the microalgae *Chlorella* sp. is very low, below even 1%. It was also observed that the addition of glycerol, while increasing the yield of microalgal biomass, did not have a significant effect on the nitrogen and phosphorus content of the biomass. However, there was a trend towards slight but decreasing nitrogen content in microalgal biomass with increasing glycerol content in the cultivation media. Under optimum conditions, we found that, for obtaining the maximum biomass (0.08 g/L nitrogen in the cultivation media and 6% glycerol), the reduction in nitrogen in microalgae biomass was about 0.99%.

## 4. Discussion

### 4.1. Application of Liquid Waste from Biofuel Production for Microalgae Cultivation

There has been a growing interest in the use of microalgae in various fields, including biofuel production [2]. However, the use of microalgae for fuel production is still being challenged due to the high material and energy costs for microalgae cultivation, resulting in high oil prices and an inability to compete with the vegetable oil normally used for biodiesel synthesis [17]. One way to reduce the cost of growing microalgae could be to replace the chemical materials used to prepare the microalgae-cultivated media by possible waste that contains required nutrients [5,6]. Such wastes containing nitrogen and phosphorus compounds are the liquid fraction after biogas production from sewage sludge and from distillers’ grain. A by-product of the production of biodiesel-technical glycerol could be used as a carbon source for algae cultivation. Its use would create a cycle in which the cultured microalgae oil would be used for biodiesel synthesis, and the technical glycerol formed during the synthesis would be returned to the microalgae cultivation. The use the waste from gaseous fuel production for the cultivation of microalgae would not only solve the problems of using liquid waste, but if the process of microalgae cultivation is incorporated into biogas production, the carbon dioxide generated by it could be successfully removed from biogas by the application of algae.

In order to reduce the cost of microalgae cultivation, studies have been carried out to replace nitrogen in the cultivation media by other sources that are cheaper than sodium nitrate, with a greater focus on the use of suitable waste, especially that obtained in liquid and gaseous biofuel production. Waste from biogas production—the liquid fraction after biogas production from sewage sludge and the liquid fraction after biogas production from distillers’ grain—were used for cultivation experiments.

The optimal nitrogen content in the cultivation media containing liquid waste from biogas production was found to be 0.08 g/L. The highest microalgae biomass concentration of 1.45 g/L was obtained when the microalgae were cultivated 22 days in media containing the liquid fraction after biogas production from distillers’ grains.

A slightly higher amount of microalgal biomass, 1.59 g/L, was obtained by growing algae in a conventional BG11 cultivation media. When the liquid fraction after biogas production from sewage sludge was used for algae cultivation, by the 22nd day of cultivation, the concentration of microalgae biomass was nearly the same as that obtained by cultivation in BG11 media, reaching 1.6 g/L.

The higher amount of liquid waste in the cultivation media resulted in a lower final concentration of microalgae biomass. The cultivation of algae in a medium with a maximum nitrogen content of 0.14 g/L from the liquid fraction after biogas production from distillers’ grain resulted in biomass concentration of only 0.75 g/L. The usage of the same amount of nitrogen from the liquid fraction after biogas production from sewage sludge for microalgae cultivation resulted in 1.1 g/L microalgae biomass concentration within 22 days.

The negative impact of the higher content of the liquid fraction in cultivation can be explained by the presence of ammonium nitrogen, which inhibits the growth of microalgae.

The negative effect of ammonium nitrogen on the accumulation of microalgal biomass has also been found by some researchers [28]. They suggest that low concentrations of NH_4_-N (up to 100 ppm) do not affect the growth rate of microalgae, whereas higher concentrations of nitrogen in the ammonium form (>200 ppm) reduce biomass yield by up to 30%. Other researchers have found that up to 100 ppm NH_4_-N does not inhibit the growth of *Scenedesmus* sp., but at high concentrations of 200–5000 ppm ammonium, biomass yield is reduced by 30% [29]. Studies by Skorupskaitė et al. showed that the yield of the microalga *Chlorella* sp. was reduced by approximately 45% in media with 576 mg/L NH_4_-N, compared to cultivation in media with ten-times-lower ammonium-N concentrations [30]. Park et al. observed similar trends when investigating the potential for ammonia removal using *Scenedesmuss* sp. microalgae. It was found that the anaerobic digestion of livestock waste in the presence of up to 100 ppm NH_4_-N did not inhibit the growth of microalgae cells, but at concentrations of 200–500 ppm NH_4_-N, the concentration of microalgae cells decreased by up to 70% [29].

The results of our performed studies show that the inhibition of microalgae growth is observed in the presence of more than 0.08 g/L total nitrogen or 0.049 g/L ammonium nitrogen in the cultivation media: biomass growth was inhibited by about 18–20%. Taken together, the results show that the liquid fraction after biogas production can be used in small quantities for the cultivation of microalgal biomass.

Park et al. found that the negative effects of ammonium nitrogen can be reduced by moderate-degree aeration of the cultivation media, which removes some of the ammonium by stripping to ammonia gas [29]. An even-better nitrogen removal efficiency from liquid waste has been found using a microalgae–bacteria consortium [31]. The microalgae–bacteria consortium for dairy wastewater remediation was reported to remove almost 100% of the nitrate and about 80% of the ammonium within 48 h, with initial nitrate concentrations ranging from 5.5 to 139 mg/L, and ammonium concentrations ranging from 16 to 23.6 mg/L. Meanwhile, Choi [32] reported that *Chlorella vulgaris* microalgae removed 85.47% of the total nitrogen from dairy wastewater within 10 days.

Summarising the results of the microalgae biomass accumulation studies on microalgae cultivation in media amended with liquid waste as a nitrogen source, it can be concluded that liquid waste from biogas production can be used for microalgae cultivation. The optimum nitrogen content in the cultivation media with liquid waste is 0.08 g/L. This medium ensures a relatively high biomass accumulation rate and a biomass yield comparable to that obtained when microalgae are grown in the conventional microalgae cultivation media BG11.

Microalgae can be grown using a mixotrophic cultivation approach, where microalgae use carbon from both inorganic and organic compounds for photosynthesis and biomass accumulation [16,26]. Heredia-Arroyo et al. found that the addition of small amounts of glucose, sucrose, or maltose to the microalgae nutrient medium increased the rate and yield of biomass accumulation [18]. A relatively high biomass concentration of 0.33 g/L was obtained with glucose [19]. Some authors have investigated the effectiveness of glycerol supplementation in microalgae cultivation, and found that the addition of 10 g/L glycerol to microalgae cultivation media can increase the growth rate of microalgae biomass by up to a factor of 6.3 [20].

In our research, the technical glycerol from biodiesel production was used as carbon source for microalgae cultivation. An increase in algae biomass yield was observed when the glycerol content in the cultivation media increased from 2 to 6%. Higher glycerol content caused a decrease in the microalgal biomass yield. At 22 days of cultivation with 6% glycerol in the cultivation media, the microalgae biomass concentration was 1.89 g/L. In this case, the addition of technical glycerol increased the biomass yield by 18.1%.

Taken together, the results suggest that the optimum concentration of technical glycerol in the cultivation media is 6%; this level allows an increase in the microalgae biomass concentration in the cultivation media from 18.1 to 20.6%, compared to cultivation without the use of technical glycerol. The use of higher levels of glycerol results in a slight decrease in algal biomass yield.

In comparison with the results of researchers using pure glycerol for microalgae cultivation, the lower technical glycerol content was found to provide the highest biomass yield. Using pure glycerol, the maximum yield was obtained at 10% glycerol in the growth cultivation media [20], while our results show that the optimal concentration of technical glycerol is 6%, and that a higher amount of technical glycerol leads to a lower microalgae biomass yield. This could be explained by the negative influence of impurities in technical glycerol (free fatty acids, methanol, soap residues) on microalgal development.

### 4.2. Elemental Composition of Microalgae Biomass

Our performed analysis of the elemental composition of microalgae biomass has shown that the elemental carbon content of the dry microalgae biomass exceeds 50%, and that it is not directly dependent on the composition of the microalgae growing medium. The nitrogen content of microalgae biomass varies between 6.98 and 7.77%. In summary, the results show that as the nitrogen concentration in the cultivation media decreases, microalgae accumulate less nitrogen in their cells; however, at the optimum nitrogen level for obtaining the highest microalgal biomass yield, when the source of nitrogen in the cultivation media is liquid waste from biogas production from distillers’ grains, the nitrogen content of the microalgal biomass is 7% lower than that in the cultivation media in the presence of nitrate nitrogen from the cultivation media BG11. The amount of phosphorus compounds accumulated in the cells of the microalga *Chlorella* sp. is very low, not even 1%. The addition of technical glycerol in the cultivation media slightly reduces the nitrogen content of microalgal biomass. In the presence of 6% technical glycerol in cultivation media, the reduction in nitrogen content was about 0.99%. This could be explained by fact that presence of phosphorus, heavy metals, and other components in the cultivation media stimulates the synthesis of non-protein compounds, such as lipids and carotenoids [33,34]. The increase in microalgae lipid content when grown in nitrogen-reduced media has been observed by a number of researchers who have investigated the potential of microalgae oil for biodiesel synthesis [35,36,37,38]. While the use of microalgae biomass for biofuel production requires a higher lipid content than protein and carbohydrates, the use of microalgae biomass for fertiliser production requires a higher nitrogen and phosphorus content. It can be finally concluded that liquid waste from gaseous or liquid biofuel production can be used as a nitrogen source for microalgae cultivation, and the conditions under which microalgae are grown can be regulated according to the intended direction of the use of the biomass.

## 5. Conclusions

In order to reduce the cost of cultivation, the source of inorganic nitrogen in the cultivation medium of the microalga *Chlorella* sp. can be replaced by the liquid waste from the production of biogas from distillers’ grain or sewage sludge. The highest microalgae biomass yield is obtained in the nutrient medium at 0.08 g/L nitrogen from liquid waste. At this nitrogen concentration, a yield of about 1.6 g/L microalgal biomass is obtained within 22 days. The by-product of biodiesel production—technical glycerol—accelerates the accumulation of microalgal biomass and increases the yield up to 20.6%. The optimum concentration of technical glycerol in the nutrient medium is 6%. The elemental carbon content of the dry microalgae biomass exceeds 50%, and is not directly dependent on the composition of the microalgae growing medium. The nitrogen content of microalgae biomass varies between 6.98 and 7.77%.

The addition of glycerol to the growth medium increases the biomass yield, but slightly reduces the nitrogen content of the algal cells. In the presence of 0.08 g/L nitrogen from liquid waste and 6% technical glycerol in the culture medium, the reduction in nitrogen content of the microalgal biomass was about 0.99%. The amount of phosphorus compounds accumulated in the cells of the microalga *Chlorella* sp. is very low, even less than 1%.

## Figures and Tables

**Table 1:** Composition of nutrient medium BG11.

Material (Producer)	Concentration in Cultivation Media
Primary Materials:	mg/L
NaNO_3_	750
K_2_HPO_4_	40
MgSO_4_·7H_2_O	75
CaCl_2_·2H_2_O	36
Citric acid	3
C_6_H_8_O_7_·xFe_3_^+^·yNH_3_	3
EDTA	1
Na_2_CO_3_	20
Microelements:	1 × 10^−3^ mg/L
H_3_BO_3_	2.86
MnCl_2_·4H_2_O	1.81
ZnSO_4_·7H_2_O	0.222
NaMoO_4_·5H_2_O	0.39
CuSO_4_·5H_2_O	0.079
Co(NO_3_)_2_·6H_2_O	0.0494

**Table 2:** Elemental composition of the microalgae *Chlorella* sp. biomass when the algae are grown in a nutrient medium supplemented with the liquid waste from the production of biogas from distillers’ grain.

Nitrogen Concentration, g/L	C, %	H, %	N, %	P, %	S, %
0.05	55.3 ± 0.31	10.05 ± 0.09	6.98 ± 0.33	0.82 ± 0.02	0.36 ± 0.04
0.08	56.7 ± 0.11	10.31 ± 0.12	7.05 ± 0.11	0.78 ± 0.02	0.33 ± 0.02
0.11	53.8 ± 0.33	9.78 ± 0.05	7.48 ± 0.08	0.85 ± 0.03	0.39 ± 0.06
0.14	56.8 ± 0.65	10.33 ± 0.06	7.67 ± 0.06	0.92 ± 0.06	0.40 ± 0.01
0.12 (control BG11)	57.2 ± 0.42	10.40 ± 0.03	7.62 ± 0.15	0.86 ± 0.01	0.37 ± 0.10

**Table 3:** Elemental composition of the microalgae *Chlorella* sp. biomass after cultivation in a nutrient medium with the addition of liquid waste after biogas production from distillers’ grain and glycerol.

Concentration of Glycerol, %	C, %	H, %	N, %	P, %	S, %
0	56.7 ± 0.11	10.31 ± 0.12	7.05 ± 0.11	0.78 ± 0.02	0.33 ± 0.02
2	55.8 ± 0.15	10.15 ± 0.11	7.09 ± 0.12	0.77 ± 0.01	0.32 ± 0.03
4	56.5 ± 0.55	10.13 ± 0.06	7.00 ± 0.08	0.88 ± 0.04	0.34 ± 0.02
6	54.9 ± 0.33	9.82 ± 0.04	6.98 ± 0.06	0.89 ± 0.02	0.37 ± 0.04
8	54.4 ± 0.17	9.66 ± 0.15	6.82 ± 0.12	0.85 ± 0.08	0.36 ± 0.01
10	52.9 ± 0.33	9.44 ± 0.08	6.89 ± 0.07	0.82 ± 0.01	0.35 ± 0.05
